# INTEREST GROUP SESSION—ENVIRONMENTAL GERONTOLOGY: PRECARIOUS AGING IN PLACE? CRITICAL PERSPECTIVES ON AGING IN CONTEXTS OF INSTABILITY

**DOI:** 10.1093/geroni/igz038.2075

**Published:** 2019-11-08

**Authors:** Jessica M Finlay, Jarmin C Yeh

**Affiliations:** 1 Social Environment and Health, Institute for Social Research, University of Michigan, Ann Arbor, Michigan, United States; 2 University of California, San Francisco, California, United States

## Abstract

Population aging and longevity in an era of immense environmental instability raises concerns about the precarity of aging and insecurity in later life. From home- and neighborhood-level insecurities to uncertainties generated by climate change or broad economic and sociopolitical upheaval across the globe, the factors contributing to instabilities relevant to older populations are heterogeneous in scale and cause. This symposium focuses on understanding older people’s needs and experiences in the context of unstable social, economic, political, and natural environments. The first paper investigates effects of socio-environmental disruption on the well-being, recovery, and resilience of older adults in Louisiana and Mississippi deeply affected by Hurricane Katrina. The second paper explores the confinement, exclusion, and loss of autonomy, as well as the creative negotiation and sociopolitical reclamation of space, among disabled older adults experiencing houselessness. The third paper discusses filmmaking with formerly homeless older adults as a method to engage marginalized individuals in community-based participatory research and better understand nuanced meanings of ‘home’. The fourth paper explores how transportation and technology can serve as both facilitators and barriers to accessibility and social connectivity among ethnically diverse low-income older adults. Altogether, the papers critically discuss novel scholarship and applied research in environmental gerontology from the micro to macro scale. The symposium inspires methodological innovation and critical research directions, and informs place-based policymaking to address diverse contexts of aging in place.

